# Retraction: Medication Non-Adherence among Patients with Heart Failure

**DOI:** 10.7759/cureus.r17

**Published:** 2019-11-05

**Authors:** Zahid U Rehman, Arsalan K Siddiqui, Musa Karim, Haris Majeed, Muhammad Hashim

**Affiliations:** 1 Miscellaneous, National Institute of Cardiovascular Diseases, Karachi, PAK; 2 Miscellaneous, Aga Khan University Medical College, Aga Khan University Hospital, Karachi, PAK; 3 Cardiology, National Institute of Cardiovascular Diseases, Karachi, PAK

This article has been retracted due to the authors' unlicensed use of The Morisky Medication Adherence Scale, otherwise known as the Morisky Scale (MMAS-8), which was copyrighted in 2006.  Additionally, the scoring and coding of the MMAS-8 is incorrect, thus invalidating the results.

